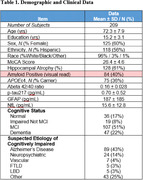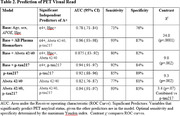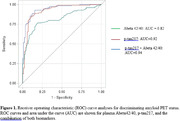# Utility of Plasma Biomarkers in Screening for Brain Amyloid in the 1Florida Alzheimer Disease Research Center (ADRC)

**DOI:** 10.1002/alz.091578

**Published:** 2025-01-09

**Authors:** Warren W Barker, Karen Velasquez, David A. Loewenstein, Rosie E Curiel, Melissa J Armstrong, Monica Rosselli, Malek Adjouadi, Breton M. Asken, David Vaillancourt, Jesse DeSimone, Glenn E. Smith, Michael Marsiske, Jacob Fiala, Darren M Weber, Jueun C Kim, Mileidys Herrera, Idaly Velez‐Uribe, Ranjan Duara

**Affiliations:** ^1^ Mount Sinai Medical Center, Miami Beach, FL USA; ^2^ University of Miami School of Medicine, Miami, FL USA; ^3^ University of Florida, Gainesville, FL USA; ^4^ Florida Atlantic University, Davie, FL USA; ^5^ Florida International University, Miami, FL USA; ^6^ Quest Diagnostics Nichols Institute, San Juan Capistrano, CA USA; ^7^ Quest Diagnostics, Mass Spectrometry R&D, San Juan Capistrano, CA USA

## Abstract

**Background:**

With the emergence of anti‐amyloid drugs, there is an increased need to determine the presence of brain amyloid in people using non‐invasive, cost‐effective biomarkers. The goal of this study was to determine the added and stand‐alone value of plasma biomarkers for predicting positive amyloid PET in a mixed sample of Hispanic and non‐Hispanic older adults.

**Method:**

Participants (n=209) from the 1Florida ADRC at Mount Sinai Medical Center, Miami Beach had neurological and neuropsychological evaluations; MRI, rated positive (Hpc+) or negative (Hpc‐) for hippocampal atrophy on visual read; amyloid PET brain scans, rated amyloid positive (A+) or negative (A‐) on visual read; blood draws for APOE ε4 genotyping (e4+, e4‐); and plasma biomarkers including Abeta 42/40 ratio (Quest Diagnostics), p‐tau217 (ALZPath), glial fibrillary acidic protein (GFAP), and neurofilament light chain (NfL) (Quanterix). A base logistic regression model including age, sex, APOEε4 status, and hippocampal atrophy was a reference to assess the value of adding plasma markers in predicting A+/A‐. Areas under the receiver operating characteristic (ROC) were computed and DeLong’s approach was used to compare ROC curves. Youden’s index was used to identify optimal sensitivities/specificities to discriminate A+ from A‐ participants.

**Result:**

Demographics and clinical data are shown in Table 1. In the base model, the AUC was 0.78 with both APOEe4 and hippocampal atrophy as significant independent predictors of A+ (Table 2). Prediction of A+ was improved by adding all plasma biomarkers (AUC=0.96; 93%/87% sensitivity/specificity), with APOEe4, Abeta42/40, and p‐tau217 as independent predictors. Adding either Abeta 42/40 (AUC=0.88) or p‐tau217 (AUC=0.94) alone to the base model also improved model prediction.

As stand‐alone predictors of A+, the optimal specific/sensitivity was 85%/89% for p‐tau217 and 77%/81% for Abeta42/40; p‐tau217 outperformed Abeta42/40 (AUC=0.92 versus 0.82) (Figure 1). There was a trend for the combination of both biomarkers to outperform p‐tau217 alone (AUC=0.94 versus 0.92).

**Conclusion:**

Positive amyloid PET status can be predicted with high accuracy in an ethnically diverse population using combinations of biomarkers, APOE genotype, and a measure of hippocampal atrophy. As a stand‐alone biomarker, p‐tau217 performed better than other biomarkers in predicting amyloid status.